# The effects of hsa-mir-26a-5p on cell proliferation, migration, and PI3K inhibitor sensitivity in metformin-resistant triple negative breast cancer cells

**DOI:** 10.55730/1300-0152.2749

**Published:** 2025-03-17

**Authors:** Şahika CINGIR KÖKER, Senem NOYAN, Banu YALÇIN, İrem DOĞAN TURAÇLI

**Affiliations:** 1 Department of Medical Biology, Faculty of Medicine, Ufuk University, Ankara, Turkiye; 2 Ogretmen Naime Tomek Research Laboratory (ONTAL), Ufuk University, Ankara, Turkiye; 3 Biotechnology Institute, Ankara University, Ankara, Turkiye

**Keywords:** Triple negative breast cancer, metformin, microRNA, PI3K inhibitor, cell migration, hsa-miR-26a-5p

## Abstract

**Background/aim:**

Metformin is commonly used to manage type 2 diabetes (T2D) and is being investigated for its potential antiproliferative effects in cancer, particularly in patients with both T2D and malignancies. Drug resistance can develop with any therapeutic agent, and metformin is no exception. As we showed in our previous study, metformin-resistant MDA-MB-468 (MET-R) cells exhibited an EMT-like phenotype. Many transcription factors, as well as miRNAs, can contribute to this altered phenotype. Our current study identifies the contribution of hsa-miR-26a-5p expression to the previously observed phenotype.

**Materials and methods:**

By utilizing bioinformatic tools, we identified hsa-miR-26a-5p, whose expression was significantly altered with increasing concentrations of metformin in MET-R cells. We rescued hsa-miR-26a-5p expression and examined the EMT phenotype and apoptotic markers via Western blot analysis.

**Results:**

We observed a reduction in hsa-miR-26a-5p expression in response to increasing concentrations of metformin in MET-R cells. Upon successful restoration of hsa-miR-26a-5p expression, a subsequent decrease in the proliferation rate was noted. Moreover, when combined with a PI3K inhibitor, we observed increased sensitivity to the PI3K inhibitor. The EMT and apoptotic markers also tended to decrease upon combinatorial treatment.

**Conclusion:**

In this study, we rescued the diminished expression of hsa-miR-26a-5p in MET-R cells to increase the sensitivity to PI3K inhibitor. The combination of a PI3K inhibitor and rescued hsa-miR-26-5p expression resulted in the restoration of the EMT phenotype and proliferation in these cells.

## 1. Introduction

Breast cancer is a multistep and heterogeneous malignancy that represents the leading cause of both incidence and cancer-related mortality among women worldwide (Polyak, 2007). In addition to environmental and genetic factors, mutations in oncogenes and tumor suppressor genes significantly contribute to the tumorigenesis of breast cancer (Osborne et al., 2004). Increasing age, menopause, high hormone levels, and obesity are among the environmental factors that account for 47% of breast cancer cases (Sieri et al., 2009; Lee et al., 2012 ; McKenzie et al., 2013 ; Wass and Finer, 2013 ).

Type 2 diabetes (T2D) is another common disease, like breast cancer, and it is also highly associated with breast cancer pathogenesis (De Bruijn et al., 2013; Hatoum and McGowan, 2015 ). It is known that the mortality rate among breast cancer patients with diabetes is higher compared to that of breast cancer patients without diabetes (De Bruijn et al., 2013). The disruption of cell metabolism connects diabetes and cancer (Glicksman et al., 1956). Hyperglycemia, characterized by elevated blood glucose levels, leads to increased insulin production—a condition known as hyperinsulinemia. Over time, this can result in the development of insulin resistance in cells. Increased insulin levels have mitogenic effects and are a significant risk factor for the development of breast cancer (Catsburg et al., 2014). Therefore, high blood glucose levels, which are the most prominent characteristic of diabetes, provide an opportunity for cancer cells to be nourished. This is why approaches such as lowering blood glucose levels are beneficial for both the treatment and prevention of cancer (Jalving et al., 2010).

Metformin, which belongs to the biguanide family, is a widely prescribed medication for diabetic patients. It is also used to manage comorbid patients with both diabetes and cancer due to its antiproliferative effects which contribute to improved survival rates in this patient population (De Bruijn et al., 2013; Zhang and Li, 2014 ). Despite studies showing a decreased cancer incidence and increased survival time in cancer patients using metformin (Zhang and Li, 2014; Xu et al., 2015a; Hou et al., 2017 ), other studies claim that no association exists between these two (Bayraktar et al., 2012; Tsilidis et al., 2014 ). Metformin primarily works by reducing hepatic gluconeogenesis and inhibiting the mitochondrial enzyme complex I of the electron transport chain, leading to a reduction in ATP production and an increase in AMP levels. Elevated AMP activates AMP-activated protein kinase (AMPK), a key regulator of cellular energy balance. Additionally, metformin administration increases insulin sensitivity in vivo, resulting in a reduction in blood glucose levels (Gonzalez-Angulo and Meric-Bernstam, 2010; Cohen and LeRoith, 2012). Moreover, elevated insulin levels are strongly associated with a higher risk of breast cancer and lower survival rates (Campagnoli et al., 2013; Noto et al., 2013 ). Therefore, the reduction of both insulin and glucose levels by metformin affects the proliferation rate of cancer cells both directly and indirectly. In the context of breast cancer, metformin reduces androgen and estrogen levels, which are strongly linked to cases occurring during the menopausal period (Campagnoli et al., 2012; Campagnoli et al., 2013 ). Therefore, metformin serves as an effective agent in reducing breast cancer risk.

In breast cancer, the PI3K/AKT/mTOR pathway plays an important role in driving cancer progression and is activated by PIK3CA (phosphatidylinositol-4,5-bisphosphate 3-kinase catalytic subunit alpha) mutations, loss of PTEN, or hyperactivity of upstream growth factor signaling (Xiao et al., 2021). PI3K inhibitors, such as alpelisib, are effective in targeting this pathway; however, therapeutic resistance occurs due to compensatory metabolic adaptations and feedback activation of survival pathways (Wright et al., 2021; Browne and Okines, 2024 ). Metformin also emerges as a promising complement to PI3K inhibitors by targeting cellular energy metabolism and suppressing mTOR signaling via AMPK activation (Dowling et al., 2007; Yue et al., 2014 ). This dual approach not only addresses the oncogenic signaling and metabolic demands of breast cancer cells but also mitigates common side effects of PI3K inhibitors, such as hyperglycemia (Shen et al., 2023). Together, these agents offer a synergistic strategy to overcome tumor growth and therapy resistance, particularly in breast cancers with metabolic vulnerabilities or pathway dysregulation. Another extensively studied aspect of metformin is its impact on epithelial–mesenchymal transition (EMT), a process commonly observed in cancer cells. EMT occurs not only during embryonic development, tissue renewal, and fibrosis, but also plays a critical role in cancer cell migration, invasion, and the development of drug resistance (Singh and Settleman, 2010; Chaffer and Weinberg, 2011 ). The anti-EMT effects of metformin have been studied in breast cancer (Vazquez-Martin et al., 2010), skin cancer (Cerezo et al., 2013), prostate cancer (Wang et al., 2023), lung cancer (Zhao et al., 2014), and thyroid cancer (Han et al., 2015), where metformin inhibited EMT. On the contrary, another study showed increased levels of EMT-related genes in silico in metformin-resistant ER (+) MCF7 cells (Oliveras-Ferraros et al., 2014). Moreover, we recently showed that metformin-resistant MDA-MB-468 (MET-R) cells exhibited a mesenchymal phenotype (Cingir Koker et al., 2022).

MicroRNAs (miRNAs) play a critical role in regulating gene expression and have emerged as key modulators in the pathogenesis of various diseases, including diabetes and cancer. Metformin treatment in T2D patients also affects miRNA profile, such that it increases the expression of miR-192 while decreasing the expression of miR-222 (Ortega et al., 2014). In this study, we investigated the effects of hsa-miR-26a-5p in MET-R cells’ proliferation and migration. Using the miRNet web-based tool (Xia Lab, University of Alberta, Edmonton, Canada), we identified this miRNA as a potential target of metformin (Chang et al., 2020). We observed reduced hsa-miR-26a-5p expression in MET-R cells compared to parental cells. After rescuing hsa-miR-26a-5p expression with miRNA mimic transfections, we observed a decreased proliferation rate in both the mimic-transfected group and the group treated with mimic and PI3K inhibitor, in a dose-dependent manner. Moreover, with scratch assay, we observed inhibition of wound closure in mimic transfected and PI3K inhibitor applied cells compared to control cells. In parallel, the expression of several EMT markers was notably reduced in the groups transfected with the mimic and treated with the PI3K inhibitor. Upon assessing sensitivity following miRNA mimic transfection, we observed increased sensitivity to the PI3K inhibitor in mimic-transfected groups compared to controls. Based on these results, we hypothesized that hsa-miR-26a-5p can act synergistically with the PI3K inhibitor in metformin-resistant breast cancer cells, resulting in decreased migration and proliferation capacity.

## 2. Materials and methods

### 2.1. Investigation of miRNA expressions in breast cancer

In this project, hsa-miR-26a-5p expression in breast cancer patients was analyzed using TCGA data (BRCA, n = 1247) via the UALCAN web-based tool (Chandrashekar et al., 2017).

### 2.2. Generation of metformin-resistant cells

Parental (a kind gift from Dr Özgür Şahin) and metformin-resistant MDA-MB-468 (MET-R) cells were cultured in a humidified atmosphere at 37 °C and 5% CO_2_ in Dulbecco’s Modified Eagle Medium (DMEM; Gibco, Thermo Fisher Scientific, Waltham, MA, USA) supplemented with 10% fetal bovine serum (FBS; Gibco, Thermo Fisher Scientific, Waltham, MA, USA), 1 mM L-glutamine, 100 U/mL penicillin/streptomycin and 1 mM sodium pyruvate (Gibco, Thermo Fisher Scientific, Waltham, MA, USA). Metformin was obtained from Merck (cat. no: 317240; Merck, Darmstadt, Germany).

Initially, 0.2 mM metformin was added to complete DMEM. The metformin concentration was doubled every 1 to 2 weeks until a final concentration of 6.4 mM was reached (Cingir Koker et al., 2022).

### 2.3. Total RNA isolation, cDNA synthesis, and RT-qPCR

Total RNA was isolated from the cell pellets using the GENEALL RNA isolation reagent (Cat. no: 305–101) and a NanoDrop spectrophotometer (Thermo Fisher Scientific, Waltham, MA, USA) was used to measure RNA purity, concentration, and integrity. RNA samples were reverse-transcribed into cDNA using the miRCURY LNA RT kit (cat. no. 339340; Qiagen, Hilden, Germany), following the manufacturer’s protocol. The concentration of each sample was adjusted to 1000 ng using RNase-free water. Following cDNA synthesis, real-time quantitative polymerase chain reaction (RT-qPCR) was performed using the hsa-miR-26a-5p miRCURY LNA miRNA PCR assay (cat. no. YP00206023; Qiagen, Hilden, Germany)), U6 snRNA miRCURY LNA miRNA PCR assay (cat. no. YP00203907; Qiagen, Hilden, Germany), and the miRCURY LNA SYBR^®^ Green PCR Kit (Qiagen, Cat. No. 339345, Hilden, Germany with the Bio-Rad CFX Connect Real-Time System (Bio-Rad, Hercules, CA, USA). Graphs were generated using the ΔΔCt method for each gene, which was normalized to the control group.

### 2.4. miRNA transfections

Cells were transfected 24 h after seeding with hsa-miR-26a-5p mimic (Qiagen, Cat. No. YM00471417, Hilden, Germany) or scrambled control (SCR), (Qiagen, YM00479902) using the HiPerFect transfection reagent (Qiagen, Cat. No. 301705, Hilden, Germany), according to the manufacturer’s protocol. The transfection efficiency was confirmed by RT-qPCR.

### 2.5. Cell viability assay

Parental and MET-R cells (5 × 10^3^) were seeded in 96-well plates, and on the following day, cells were treated with miRNA mimic, SCR, and various doses of PI3K inhibitor LY294002 (Cell Signaling Technology-CST, 9901P). At the end of either 24 or 48 h, the MTT assay (3-(4,5-dimethyltiazol-2-yl)-2,5-diphenyltetrazolium bromide; Merck, 475989) was performed according to the manufacturer’s instructions. Color absorbance was measured using microplate reader (SpectraMax iD3,Molecular Devices, San Jose, CA, USA) at a wavelength of 570 nm. Results were normalized against the mean measurements from at least four replicates.

### 2.6. Western blot

At the end of 1 week for each indicated concentration, cell pellets were collected from cells which were seeded 2 × 10^5^ per 6-well plate. Proteins were isolated from these pellets using freshly prepared LSB buffer containing protease inhibitor cocktail (CST, 5871S) and phosphatase inhibitor cocktail (CST, 5870S). The BCA protein assay reagent kit (Thermo Scientific, Cat. No. 23227, Waltham, MA, USA) was used to determine total protein concentrations. Before gel loading, proteins were denatured with 4X loading dye (Bio-Rad, Cat. No. 161–0747, Hercules, CA, USA) at 95 °C for 5 min. Proteins were separated by 10% SDS-PAGE (Bio-Rad, Cat. No. 161–0183, Hercules, CA, USA) and transferred onto PVDF membranes (Merck Millipore, Cat. No. 3010040001, Darmstadt, Germany) using the semidry transfer method. Membranes were then blocked with 5% milk prepared in Tris-buffered saline containing 0.2% Tween-20 (TBST) for 1 h at room temperature. Membranes were incubated overnight at 4 °C with the following primary antibodies at a 1:1000 dilution, unless otherwise specified. Membranes were washed three times with TBST for 10 min and then incubated with secondary antibodies (1:5000) for 1 h at room temperature. Membranes were washed again three times with TBST for 10 min. Protein bands were detected by using ECL reagent (Amersham, RPN2232), and images were captured using Bio-Rad ChemiDoc XRS+ system. GAPDH served as the loading control. The antibodies used in this study included GAPDH (Cell Signaling Technology, Cat. No. 2118, Danvers, MA, USA), SLUG (Cell Signaling Technology, Cat. No. 9585, Danvers, MA, USA), ZEB1 (Cell Signaling Technology, Cat. No. 3396, Danvers, MA, USA), VIMENTIN (Cell Signaling Technology, Cat. No. 5741, Danvers, MA, USA), BAX (Cell Signaling Technology, Cat. No. 2772, Danvers, MA, USA), BCL-XL (Cell Signaling Technology, Cat. No. 2762, Danvers, MA, USA), E-cadherin (Cell Signaling Technology, Cat. No. 3195, Danvers, MA, USA), and p-P65 (Cell Signaling Technology, Cat. No. 3033, Danvers, MA, USA).

### 2.7. Wound healing assay

To assess the migration capacity of MET-R cells transfected with either miRNA mimic or SCR, 2 × 10^5^ cells were seeded in 6-well plates. On the following day, vertical wounds were introduced using a 200 μL sterile pipette tip, and images were captured using an Olympus CKX53 microscope (Olympus, Tokyo, Japan). Images were also captured at 24, 48, and 72 h. At least six images were taken at each time point. The distance between the two wound edges was measured in a blinded manner using the Olympus cellSens Entry software (Olympus, Tokyo, Japan).

### 2.8. Statistics

Graphpad (Prism 8, GraphPad Software, San Diego, CA, USA) was used to plot graphs and for statistical analysis. For RT-qPCR, log_2_ of ΔΔCt values were plotted and analyzed using one-way ANOVA with multiple comparisons (Dunnett’s test). MTT results were plotted as relative percentages of cell viability compared to the control group and analyzed using two-way ANOVA with multiple comparisons (Tukey’s test). For the wound healing assay, graphs were generated using relative migration percentages compared to the control group and analyzed using two-way ANOVA with multiple comparisons (Sidak’s test).

## 3. Results

### 3.1. Hsa-miR-26a-5p expression is low in breast cancer samples and higher expression is related with good prognosis

**Figure 1:**
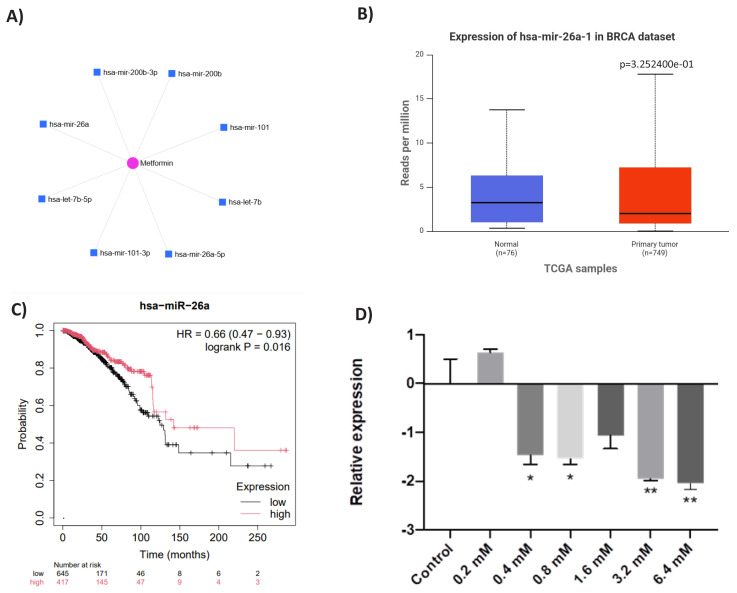
Investigation of hsa-miR-26a-5p expression in breast cancer patients and cells. Hsa-miR-26a-5p appears as one of the targets of metformin **(A)**. According to the TCGA dataset, the expression of hsa-miR-26-5p is lower in tumor samples compared to corresponding normal samples **(B)**. Kaplan–Meier survival analysis of hsa-miR-26a-5p showed that its high expression is significantly correlated with good prognosis **(C)**. With increasing doses of metformin, the expression of hsa-miR-26a-5p decreased, with the greatest reduction observed in 6.4 mM MET-R cells. The expression of hsa-miR-26a-5p was normalized against U6 snRNA expression **(D)**.

In our previous study, we observed increased metastatic behavior and EMT-like phenotypic changes in MDA-MB-468 cells with increasing levels of metformin resistance (Cingir Koker et al., 2022). To identify the underlying epigenetic factors of this resistance mechanism, the miRNet software was used. Using this software, which efficiently identifies, categorizes, and predicts miRNAs affected by specific agents, we identified hsa-miR-26a-5p as one of the miRNAs most affected by metformin (Figure 1A). When the expression of this miRNA was examined in the TCGA dataset, it was found to be lower in primary tumor samples compared to normal samples (Figure 1B). Moreover, Kaplan–Meier survival analysis revealed that higher hsa-miR-26a expression is associated with better prognosis (Figure 1C). Next, when the expression pattern of this miRNA was examined across various concentrations of metformin-resistant (MET-R) cells, hsa-miR-26a-5p expression significantly decreased with increasing resistance, except at 0.2 mM metformin (Figure 1D).

### 3.2. Effect of restored hsa-miR-26a-5p on cell proliferation and PI3K inhibitor sensitivity in MET-R cells

Since increased hsa-miR-26-5p expression was associated with better prognosis and its levels were lower in highly resistant MET-R cells, we aimed to restore its expression using a miRNA mimic. We selected 6.4mM MET-R cells for mimic transfection, as these cells exhibited the lowest expression of hsa-miR-26a-5p.

**Figure 2:**
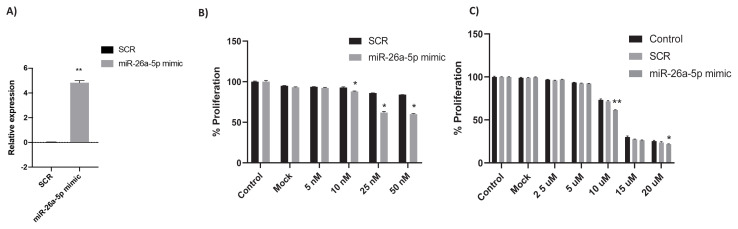
Mimic hsa-miR-26a-5p was tested and a fivefold increase was observed in 6.4 mM MET-R cells, the expression of hsa-miR-26a-5p was normalized against U6 snRNA expression **(A)**. The effects of different SCR and hsa-miR-26a-5p transfection concentrations on cell viability of MET-R cells were assessed **(B)**. The proliferative effects of various LY294002 concentrations on cell viability of control, SCR and hsa-miR-26a-5p transfected MET-R cells **(C)**. The significance demonstrated between SCR and hsa-miR-26a-5p (*) p < 0.05, (**) p < 0.0001.

First, the hsa-miR-26a-5p mimic was tested in 6.4 mM MET-R cells. We achieved a fivefold increase in its expression using a 25 nM miRNA mimic (Figure 2A). To determine the effects of varying miRNA mimic concentrations on cell proliferation, 6.4 mM MET-R cells were transfected with 5, 10, 25, or 50 nM hsa-miR-26a-5p mimics or SCR for 48 h. Cell proliferation decreased significantly with 10, 25, and 50 mM hsa-miR-26a-5p mimics compared to SCR (Figure 2B). To assess the effect of hsa-miR-26a-5p on sensitivity to the PI3K inhibitor LY294002, both the mimic and LY294002 were coadministered at various doses, with 25 nM miRNA mimic used in each case. As shown in Figure 2C, sensitivity to LY294002 significantly increased at 10 and 20 μM when combined with hsa-miR-26a-5p mimic in 6.4 mM MET-R cells.

### 3.3. Effect of restored hsa-miR-26a-5p and PI3K inhibitor combination on migration of MET-R cells

**Figure 3:**
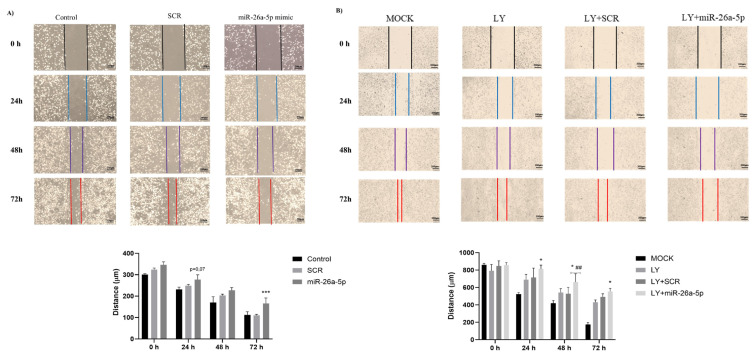
Effect of hsa-miR-26a-5p restoration on migration of MET-R cells. The representative images of control, SCR, and hsa-miR-26a-5p transfected MET-R cells show wound closure over time. Hsa-miR-26a-5p mimic significantly decreased migration capacity of MET-R cells. (***) p < 0.001 hsa-miR-26a-5p mimic compared to SCR **(A)**. Hsa-miR-26a-5p mimic significantly decreased migration in LY-treated MET-R cells. (*) p < 0.05 LY+miR-26a compared to LY, (##) p < 0.01 LY + MIMIC compared to LY + SCR **(B)**. The representative images of Mock, LY, LY + SCR, and LY + miR-26a-5p mimic transfected MET-R cells showing wound closure over time. Images were taken with 4× objective.

Given that an EMT-like phenotype and increased metastatic capacity were previously observed in MET-R cells (Cingir Koker et al., 2022), we aimed to assess whether restoration of hsa-miR-26a-5p could directly affect the migration capacity of these cells. To investigate this, a scratch assay was performed on these cells. At 48 and 72 h, hsa-miR-26a-5p mimic significantly reduced the migration capacity of MET-R cells compared to SCR, as the wound closure distance was shorter in mimic-transfected cells than in control cells (Figure 3A). Moreover, combining miRNA mimic transfection with the PI3K inhibitor LY294002 led to a more pronounced reduction in migration capacity at 24, 48, and 72 h compared to other groups (Figure 3B).

**Figure 4:**
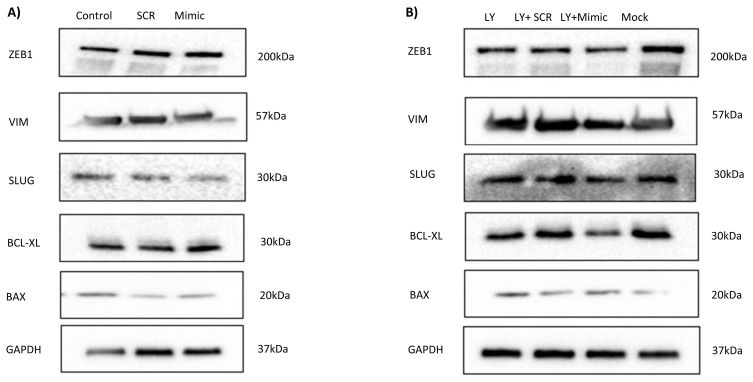
Effect of hsa-miR-26a-5p restoration and LY294002 on protein expressions in MET-R cells. Expression levels of ZEB1, VIM, SLUG, BCL-XL, BAX, and GAPDH in only mimic transfected group **(A)** and in mimic and LY294002 combination group **(B)**.

To further investigate the effect of hsa-miR-26a-5p mimic on protein expression in MET-R cells, Western blot analysis was performed. Compared with SCR, hsa-miR-26a-5p transfection reduced SLUG expression and increased BAX and BCL-XL levels, thereby slightly increasing the BAX/BCL-XL ratio at 48 h. However, ZEB1 and VIMENTIN expression levels did not change following transfection (Figure 4A, Figure S1). Combination of mimic transfection with LY294002 resulted in reduced SLUG and VIMENTIN expression. Furthermore, in this combined setting, BAX expression increased while BCL-XL expression decreased, leading to a higher BAX/BCL-XL ratio compared to the SCR group (Figure 4B, Figure S1). Moreover, p-p65 expression was higher in the miR-26a mimic group compared to the SCR group. In contrast, p-p65 levels were lower in the LY294002+miR-26a mimic compared to LY294002 + SCR group. This clearly correlated with the reduced migration and proliferation rates observed in these groups. Additionally, E-cadherin levels were increased in the mimic and LY+ mimic groups, consistent with the wound healing assay results and supporting reduced migration capacity (Figure S2).

## 4. Discussion

Numerous studies have demonstrated that metformin contributes to the inhibition of tumor progression (Buzzai et al., 2007; Algire et al., 2010 ; Blandino et al., 2012 ; Galal et al., 2024 ). Despite its well-documented anticancer effects, several studies have indicated that cancer cells may develop reduced sensitivity and increased resistance to metformin (Cingir Koker et al., 2022; Seo et al., 2023 ).

Drug resistance in cancer is a complex and multifactorial phenomenon involving newly acquired mutations and amplifications in tumor suppressor genes and oncogenes, altered drug uptake and efflux, enhanced drug metabolism, increased DNA repair, epigenetic modifications, suppression of apoptosis and autophagy, EMT, tumor microenvironmental changes, heterogeneity, and cancer cell plasticity (Haider et al., 2020; Nussinov et al., 2021 ). The mechanisms underlying metformin resistance in cancer cells remain unclear. This resistance may be driven by mutations in mTOR or AMPK signaling pathways. For instance, AMPK dysfunction or mutations in mTOR may reduce the drug’s efficacy. Multidrug resistance (MDR) proteins, such as P-glycoprotein, which actively expel drugs from the cells, can be overexpressed in certain cancer cells (Tian et al., 2023). Conversely, elevated insulin levels resulting from hyperglycemia or insulin resistance may activate growth-promoting pathways such as PI3K/AKT (Varma et al., 2005), thereby counteracting metformin’s inhibitory effects on cancer cells. Metformin’s known mechanism includes reducing glucose availability to tumor cells (Repas et al., 2023); however, its efficacy may be compromised if tumor cells adapt to alternative metabolic pathways, such as relying on fatty acid oxidation.

Previously, we investigated three widely studied breast cancer cell lines—MCF7, MDA-MB-231, and MDA-MB-468—with respect to their metastatic behavior under long-term metformin treatment. We observed changes only in MDA-MB-468 cells, which exhibited increased migration capacity and a shift towards a mesenchymal-like phenotype with increasing doses of long-term metformin treatment. Moreover, RNA and protein expression profiles supported an EMT-like phenotype, indicating that metformin’s effects depend on breast cancer subtype and should be carefully evaluated before administration (Cingir Koker et al., 2022). In addition to epigenetic factors, the miRNA expression profile also impacts the development of MDR (An et al., 2017). As reported in the literature, numerous miRNAs show altered expression in drug-sensitive compared to drug-resistant cancer cells (Fojo, 2007). For example, upregulation of miR-138 significantly downregulates ABCB1 expression, thereby reversing Adriamycin resistance in the MDR HL-60/VCR leukemia cell line (Zhao et al., 2010). In breast cancer cells, miR-298 can downregulate P-GP expression by directly binding to its 3′UTR, thereby reversing doxorubicin resistance (Bao et al., 2012).

It has been shown that hsa-miR-26a-5p is dysregulated in many cancer types, as it is located in a fragile chromosomal region (3p23) (Calin and Croce, 2006). It has been shown that hsa-miR-26a plays a critical role in a variety of cellular processes, including proliferation, apoptosis, and the cell cycle. As previously noted, its reduced expression is also associated with a poor prognosis. Furthermore, hsa-miR-26a-5p has the potential to alter tumor responsiveness to chemotherapy, indicating that the combination of hsa-miR-26a-5p with chemotherapeutic agents could serve as a novel approach for cancer treatment. For example, in pancreatic cancer, increased expression of hsa-miR-26a-5p has been demonstrated to enhance drug retention and boost responsiveness to gemcitabine (Batchu et al., 2015). VEGFA, a key proangiogenic factor, is modulated by hsa-miR-26a-5p through pathways such as PI3K/Akt/HIF-1α, influencing tumor angiogenesis in glioma and hepatocellular carcinoma (HCC) (Chai et al., 2013; Qian et al., 2013 ). In addition, hsa-miR-26a-5p inhibits angiogenesis in HCC by targeting PIK3C2α and HGF-related pathways. Based on this, it can be postulated that combining miR-26a mimics with PI3K inhibitors can enhance antitumor activity.

In this study, we aimed to rescue the downregulated hsa-miR-26a levels in MET-R cells by using a miRNA mimic, in order to reverse the EMT phenotype observed in our previous study (Cingir Koker et al., 2022). Furthermore, we tested the sensitivity of these cells to a PI3K inhibitor in combination with the hsa-miR-26a-5p mimic. After successful restoration of hsa-miR-26a-5p in MET-R cells, we observed a decreased proliferation rate in the restored MET-R cells. When combined with a PI3K inhibitor, we observed increased sensitivity to the inhibitor upon restoration of hsa-miR-26a levels. Migration capacity as well as EMT marker expression was reduced upon hsa-miR-26a-5p restoration, and this effect was even more pronounced when combined with a PI3K inhibitor.

The PI3K pathway is one of the most widely studied pathways in breast cancer, as it plays a significant role in the proliferation and EMT of cancer cells (Paplomata and O’Regan, 2014; Xu et al., 2015b). For example, chemokines are involved in EMT progression through the PI3K/AKT signaling pathway, such that coactivation of CCL20 and CXCL8 results in upregulation of VIMENTIN, SNAIL, and CDH2 levels to promote EMT (Shen et al., 2019). In ovarian cancer cells, it has been demonstrated that fibroblast growth factor (FGF2) results in increased expression of SLUG and ZEB1 by activating the PI3K/AKT/mTOR and MAPK/ERK signaling pathways (Lau et al., 2013). In another study, insulin-like growth factor-1 (IGF-1) was shown to induce SLUG and SNAIL expression via the PI3K/AKT signaling pathway (Lau and Leung, 2012).

The markers of EMT are affected by transcription factors as well as miRNA signaling (Moyret-Lalle et al., 2014). Among miRNAs, hsa-miR-26a-5p expression was significantly downregulated in tumor tissue, suggesting that this low expression of hsa-miR-26a-5p resulted in the upregulation of vimentin and the downregulation of E-cadherin, which eventually promoted EMT (Chang et al., 2017). In another study, it was proposed that hsa-miR-26a-5p regulates PTEN expression, which eventually affects the downstream PI3K/AKT pathway (Tian et al., 2013).

The PI3K/AKT signaling pathway contributes to chemoresistance in cancer by promoting antiapoptopic proteins such as BCL-XL and BCL-2, while inhibiting proapoptotic such as BAX. This imbalance favors cell survival through mechanisms such as the phosphorylation and cytoplasmic retention of BAD (Rascio et al., 2021).

Considering the above-mentioned phenomenon, our initial objective in this study was to rescue hsa-miR-26a-5p expression in MET-R cells, since increasing resistance doses were associated with decreased hsa-miR-26a-5p expression. In this way, we aimed to utilize the effects of restored hsa-miR-26a-5p expression, which exerts inhibitory effects on the PI3K/AKT pathway. This eventually altered the EMT phenotype of MET-R cells. We further observed a pronounced decrease in the EMT profile of MET-R cells when hsa-miR-26a-5p mimic expression was combined with a PI3K inhibitor. This combination disrupts survival mechanisms and enhances apoptosis, suggesting its therapeutic potential in cancer treatment.

Given their critical roles in regulating multiple pathways involved in therapy resistance, miRNAs have emerged as potential biomarkers for predicting therapeutic outcomes and as targets for novel therapeutic strategies aimed at overcoming resistance in cancer treatment. As demonstrated in our study, the expression of hsa-miR-26a-5p is significantly altered in the presence of metformin, a compound that is not typically classified as a chemotherapy drug. This change in miRNA expression plays a crucial role in modulating the cellular response to the drug. Our findings underscore the necessity of further investigation into this phenomenon, particularly in animal models and human tissues, to better understand its implications in drug response. This approach presents scientifically valuable insights that could have broader therapeutic implications.

## Supplementary material

Figure S1 The quantification of protein expressions normalized against GAPDH, in only mimic transfected group (A) and in mimic and LY294002 combination group (B).

Figure S2 Effect of hsa-miR-26a-5p restoration and LY294002 on protein expressions in MET-R cells. Expression levels of p-P65, E-CADHERIN, and GAPDH in only mimic transfected group (A) and in mimic and LY294002 combination group (B).
