# Supplementary figures and images for: The effects of hsa-mir-26a-5p on cell proliferation, migration, and PI3K inhibitor sensitivity in metformin-resistant triple negative breast cancer cells
Source: Turk J Biol. 2025 Mar 17;49(3):336–46. doi: 10.55730/1300-0152.2749 (PMC12266353; doi:10.55730/1300-0152.2749)

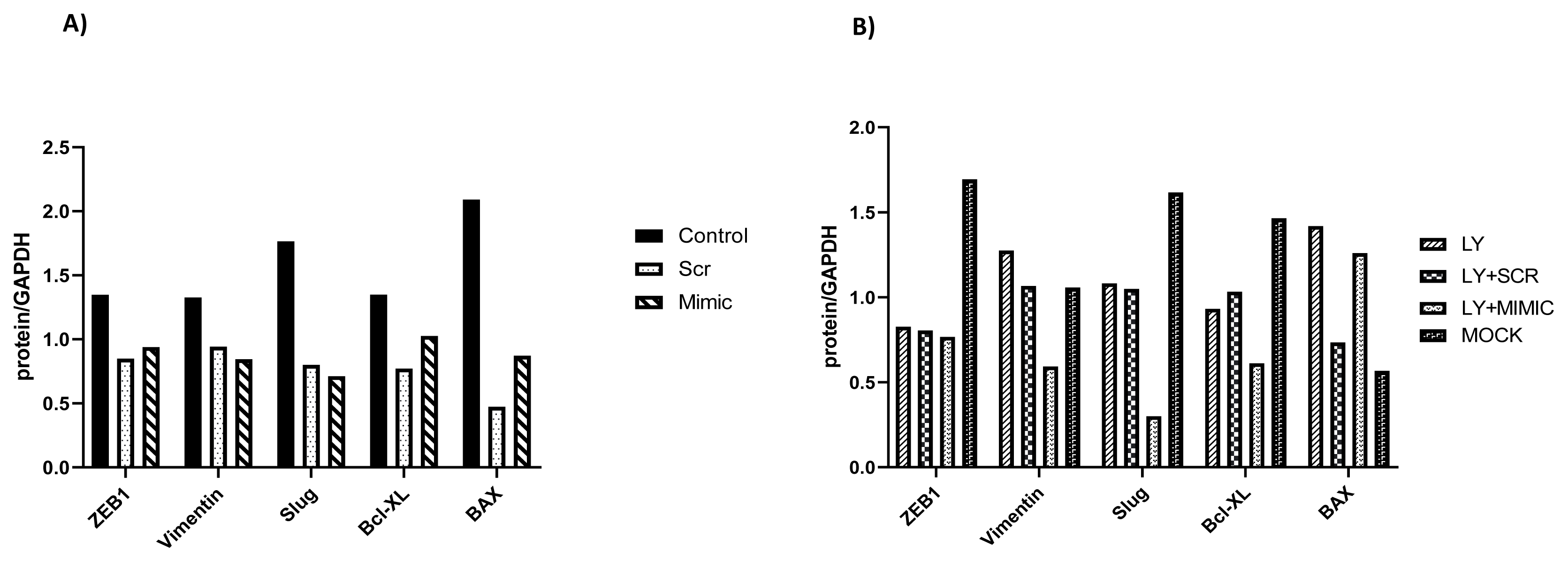

Supplement: Figure S1 — The quantification of protein expressions normalized against GAPDH, in only mimic transfected group (A) and in mimic and LY294002 combination group (B). [file tjb-49-03-336s1.tif]

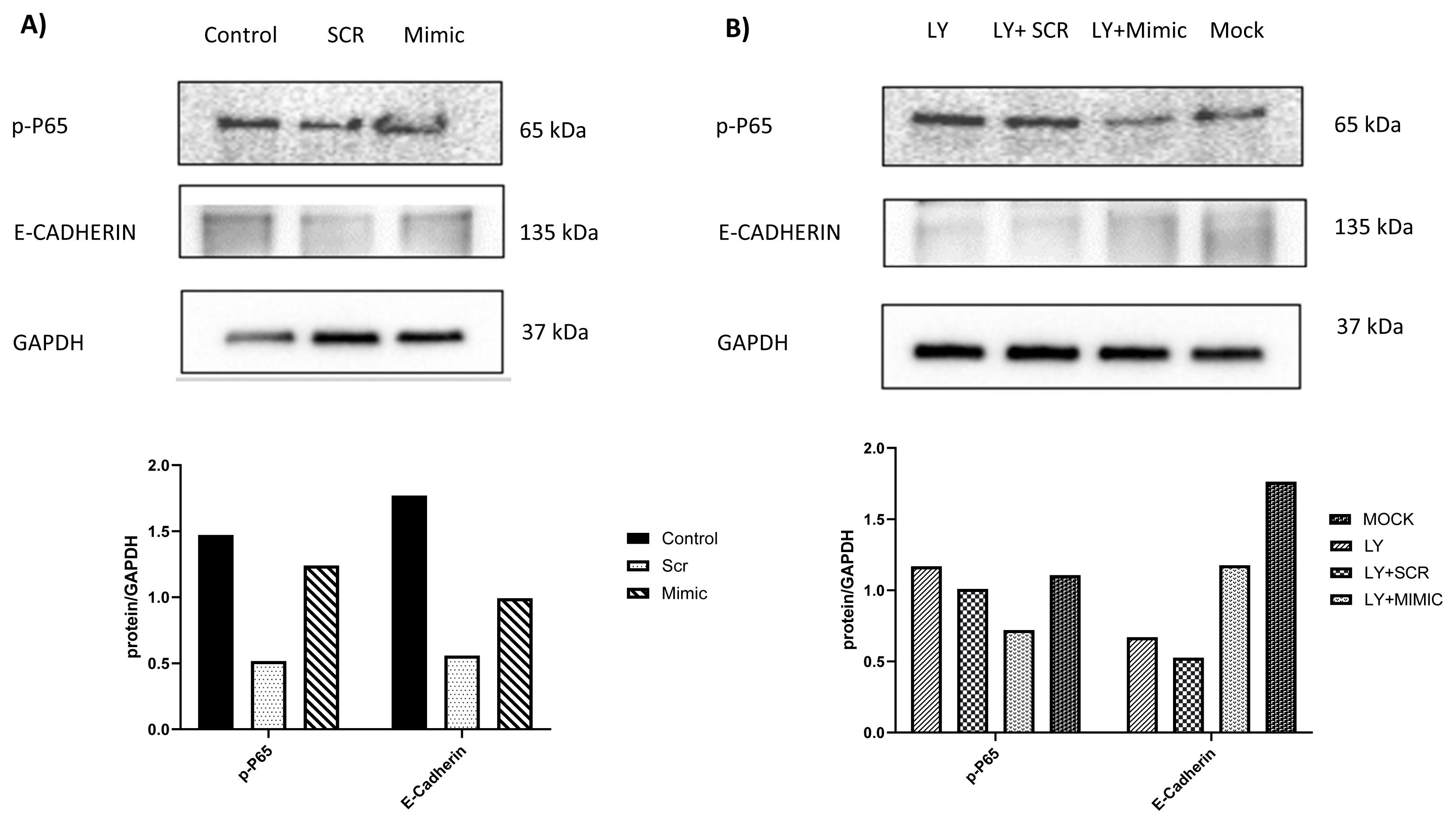

Supplement: Figure S2 — Effect of hsa-miR-26a-5p restoration and LY294002 on protein expressions in MET-R cells. Expression levels of p-P65, E-CADHERIN, and GAPDH in only mimic transfected group (A) and in mimic and LY294002 combination group (B). [file tjb-49-03-336s2.tif]
